# Echoes of the Hot Springs Meeting

**Published:** 1895-01

**Authors:** 


### ECHOES OF THE HOT SPRINGS MEETING.

The Journal has received the following notes, which explain themselves and to which we gladly give space :

Hot Springs, Ark., Nov. 30, 1894.

*Dr. W. W. Potter, Editor, Buffalo, N. Y.:*

Dear Doctor—I beg space in the valuable columns of your journal to thank the doctors, their wives and friends for the beautiful present they so generously remembered me with. Yours truly,

MRS. LYMAN T. HAY.

The acknowledgment is as follows :

On November 23d the wheels and flying sparks took from me the most enthusiastic and charming assembly of people it has ever been my good fortune to know, and after many miles lay between us I received an exquisite token—a circular pin, of richest gold, with a diamond in the center. Its rays showed me plainly the brilliancy of the givers. I do not thank them *four or five times*, but four or five million times.

Yours appreciatively,

LOTAWANNA F. HAY.

---

The Hon. William H. Martin, of Hot Springs, made one of the cleverest addresses in welcoming the Mississippi Valley Medical Association to that city to which we ever listened. Seldom does it fall to the lot of a layman and particularly a lawyer to address a company of doctors without laying himself open to criticism in some particular. But Mr. Martin in his address made no mistake. He captured his audience at the outset and held it to the end.

We wish we had space for the address in full, but can only print his closing sentences, which form a peroration of exceptional merit and one that deserves preservation in the memory of all physisians.

I am reminded, Mr. President, that my office is to bid you welcome to Hot Springs, and the welcome that I voice will surely be exemplified by cordial treatment during your stay. Just what necessity there may be for extending a formal welcome to our friend, the doctor, I do not clearly see, for if there be a man in the wide world who is welcome at all times and places, whether his visit be professional, or social it is he. It is so at every stage of life. In the hour of tribulation that precedes our advent into this world of beauty, of brightness, and of love, when more than one life lies trembling in the hands of fate, it is into the face of our good friend the doctor, that we all look for comfort, and whose expression will stamp its impress upon our own.

Or, if it be when life's fitful fever is over, and the sands of time have run their course, his kindly face is with us still, and his office is at once to administer relief to the dying, and comfort to those who remain.

God bless the educated, kind-hearted, sensible doctor! May the judgment pronounced upon his life's work be as welcome to him as his kindly presence always is to us.

---

The railroad facilities to and from Hot Springs were exceptionally good, as we mentioned in our December issue. Besides the efforts of Col. Townsend, of the Iron Mountain Route, it should not be forgotten that Mr. W. F. Snyder, general agent of the Big Four at St. Louis, was especially active in providing for the comfort of the travelers from the East.

It does not often fall to the lot of a great railway to be represented by a more courteous official than Col. Snyder proved to be. Under his keen and watchful eye no detail of comfort was omitted, and everything that could contribute to safe and rapid transit was provided. It is a pleasure to ride over such a route as the Big Four.

---

Among other interesting features was the action of the association on the recommendations contained in the president's address. The ethical question was disposed of as follows: . . . . . . . .
VII. This association believes that in the proper education of physicians by adequate preliminary training, a medical college curriculum of four years and a final examination for license by the state lies a true solution of many, if not all the much controverted questions of ethics; and that in the present day a further agitation of these questions by debate on the floor of societies met together for scientific advancement is unwise and injudicious. . . .

The above recorded action is respectfully commended to the notice of the American Medical Association as a model for its future guidance.

---

One of the pleasant episodes of the convention occurred during the annual banquet which was held on Thursday evening, November 22d, at the Park Hotel. During the midst of the speech-making, when everything was at high water mark, under the masterly superintendence of Dr. I. N. Love, that prince of toast-masters, he called a halt and for a moment expectation stood agog wondering what was going to happen.

Taking advantage of the momentary silence, Dr. W. H. Daly, of Pittsburg, in an appropriate speech, presented to Col. H. C. Townsend, general agent of the Iron Mountain Route, who was personally conducting the tour, a beautiful jewelled scarf pin. A like office was performed for Col. Charles E. Ware, general advertising agent of the Missouri Pacific Railway, by Dr. Love. The tourists contributed the money for these two beautiful jewels, and they were procured in St. Louis by John W. Cox, Esq., vice-president of the Antikamnia Chemical Co., whose kind offices in that regard the contributors desire to acknowledge.

